# Implementation of 5S management method for lean healthcare at a health center in Senegal: a qualitative study of staff perception

**DOI:** 10.3402/gha.v8.27256

**Published:** 2015-04-07

**Authors:** Shogo Kanamori, Seydou Sow, Marcia C. Castro, Rui Matsuno, Akiko Tsuru, Masamine Jimba

**Affiliations:** 1 Department of Community and Global Health, Graduate School of Medicine, University of Tokyo, Bunkyo-ku Tokyo, Japan; 2 Takemi Program in International Health, Harvard T. H. Chan School of Public Health, Harvard University, Boston, MA, USA; 3 IC Net Limited, Saitama, Japan; 4 Agence Africaine de Santé Publique, Dakar, Senegal; 5 Department of Global Health and Population, Harvard T. H. Chan School of Public Health, Harvard University, Boston, MA, USA; 6 Faculty of Medicine School of Health Sciences, Gunma University, Maebashi, Gunma, Japan

**Keywords:** 5S, lean, healthcare, quality improvement, intervention programs, work environment, qualitative study

## Abstract

**Background:**

5S is a lean method for workplace organization; it is an abbreviation representing five Japanese words that can be translated as *sort*, *set in order*, *shine*, *standardize*, and *sustain*. The 5S management method has been recognized recently as a potential solution for improving the quality of government healthcare services in low- and middle-income countries.

**Objective:**

To assess how the 5S management method creates changes in the workplace and in the process and outcomes of healthcare services, and how it can be applicable in a resource-poor setting, based on data from a pilot intervention of the 5S program implemented in a health facility in Senegal.

**Design:**

In this qualitative study, we interviewed 21 health center staff members 1 year after the pilot intervention. We asked them about their views on the changes brought on by the 5S program in their workplace, daily routines, and services provided. We then transcribed interview records and organized the narrative information by emerging themes using thematic analysis in the coding process.

**Results:**

Study participants indicated that, despite resource constraints and other demotivating factors present at the health center, the 5S program created changes in the work environment, including fewer unwanted items, improved orderliness, and improved labeling and directional indicators of service units. These efforts engendered changes in the quality of services (e.g. making services more efficient, patient-centered, and safe), and in the attitude and behavior of staff and patients.

**Conclusions:**

The pilot intervention of the 5S management method was perceived to have improved the quality of healthcare services and staff motivation in a resource-poor healthcare facility with a disorderly work environment in Senegal. Quantitative and qualitative research based on a larger-scale intervention would be needed to elaborate and validate these findings and to identify the cost-effectiveness of such intervention in low- and middle-income countries.

5S stands for five Japanese words, *Seiri*, *Seiton*, *Seisou*, *Seiketsu*, and *Shitsuke*, which broadly refer to maintaining cleanliness. These five words, often translated in English as *sort*, *set in order*, *shine*, *standardize*, and *sustain*, represent a set of practices for improving workplace organization and productivity (1–4) . The 5S management method is recognized as the foundation of lean healthcare approaches, which maximize value-added levels by removing all factors that do not generate values (5). It evolved in manufacturing enterprises in Japan, and it was introduced to the manufacturing sector in the West in the 1980s (2). It has now been applied to the healthcare sector as a systematic method of organizing and standardizing the workplace for lean healthcare (6), and it has been recognized as a low-cost, technologically undemanding approach that serves as a starting point for the improvement of healthcare services (3, 6–9) .

The 5S management method has been suggested recently as a method for quality improvement of government healthcare services, particularly in low- and middle-income countries. The governments of Sri Lanka and Tanzania have officially adopted 5S as a national strategy for healthcare service quality improvement (10, 11). In Senegal, 5S was introduced to the healthcare sector under a pilot intervention program of the Japan International Cooperation Agency (JICA) in 2007 (12). Based on experiences gained through the pilot intervention, the JICA-assisted Project for Reinforcement of the Health System in Senegal (Projet d'Appui au Renforcement du Système de Santé au Sénégal, or PARSS) was initiated in 2011. It aimed at establishing a 5S intervention model to address common chronic problems in the work environment of health centers, such as a lack of orderliness with documents and supplies, deficient labeling and directional indicators of service units, and precarious overall cleanliness (13). The implementation of PARSS resulted in the inclusion of 5S in the national strategy for improving the quality of healthcare services (13, 14).

The impact of the application of the 5S management method in the healthcare sector has been documented in the United States (15–18) , India (19), Jordan (20), and Sri Lanka (21), although other lean tools and methods were often combined with the 5S management method. Observed changes as a result of these interventions included improved working processes and increased physical space (16, 18–20) , elimination of safety violations and improved compliance with regulations (15), improved clinical indicators of safety (21), and increased time with patients and improved patient satisfaction (17).

Despite these findings, little is known about the specifics of how the 5S management method changes the quality of healthcare services. Furthermore, no study has focused on its application in a resource-poor setting. Several studies targeted hospitals in low- and middle-income countries and identified measurable changes resulting from the 5S management method, such as improved process flows, increased capacity, and shorter stays for all patient classes at an emergency department (19); potential reductions in the drug-dispensing cycle time at an inpatient pharmacy (20); and reductions in the infection rate post Caesarean section and in the stillbirth rate (21). However, they did not note explicitly that the studied facilities faced resource constraints.

To address these issues, we conducted a qualitative study to explore how the 5S management method pilot intervention created changes in the workplace and in the process and outcomes of healthcare services. We also explored if the method was applicable in a healthcare facility facing resource constraints. This article provides insights into the potential applicability of the 5S management method to government healthcare facilities in low- and middle-income countries.

## Methods

### Target facility

The health center where the qualitative study was conducted is located in the Tambacounda region, which is 462 km away from Dakar, the capital of Senegal. At the kick-off meeting of PARSS, conducted in May 2011 in Tambacounda, project stakeholders reached the consensus to select it as the facility at which to start the 5S management method before expansion of the method to other health centers. The reason behind this selection was primarily associated with ease of physical access by the project stakeholders who would participate in the pilot intervention. At the time of the study, the health center had 78 staff members and a range of service units and offices, including outpatient consultation, maternity, dental, pediatric, immunization, laboratory, social counseling, health education, and nutrition programs; a pharmacy; inpatient wards; and administrative offices. The health center is located in a poverty-stricken area that is characterized by comparatively lower healthcare service and economic indicators than other areas of Senegal; the percentage receiving antenatal care from a skilled provider is 79% in Tambacounda, whereas the country average is 93%, and 52.9% of the population in Tambacounda fall in the first economic quintile of the country average (22).

### Pilot 5S intervention

The implementation of the 5S management method pilot intervention (hereinafter referred to as the *5S program*) was conducted under the JICA-assisted project, PARSS, and involved three phases: 1) training and planning for the application of the 5S management method, 2) 5S practices at each unit, and 3) progress monitoring. The ultimate objectives of PARSS were to standardize activities involved in these three phases and to integrate those into the health system's administrative process to be managed by government officials at the national and regional levels. However, because this was the initial experimental intervention, all activities were facilitated by PARSS team members consisting of foreign experts on 5S and Senegalese government officials who had prior experiences in 5S practice elsewhere. Prior to initiating the activities, PARSS team members visited all the locations in the health center to obtain insights into the baseline situation.

Phase 1 consisted of a 1-day workshop for training and planning for the application of the 5S management method; it was conducted in July 2011 at the health center with the support of PARSS team members. Sixty-two staff members of the health center, representing all the clinical, administrative, and support staff available on the day of the event, attended the program. None of the staff had prior exposure to 5S practice. The workshop program consisted of lectures and practical sessions. In the lecture session, a series of presentations were made on the principles of 5S and its applications in a healthcare facility. During the practical sessions, several service units and offices were bundled into the same category according to physical arrangement and proximity, and the health center premises were divided into nine locations: 1) administration office; 2) primary healthcare supervisor's office and social worker's office; 3) laboratory, drug store, and ticket counter; 4) dental unit; 5) health education unit; 6) pediatric unit, expanded program on immunization (EPI) unit, elderly support office, and nutrition center; 7) outpatient medical clinic; 8) supply manager's office and outside areas; and 9) maternity unit. Staff members were divided into nine groups, each assigned to one of the nine locations closely related to their job duties. Participants visited their assigned areas, conducted situation analyses, and developed action plans for improvements in accordance with the 5S criteria. The subsistence allowance was paid to all participants in accordance with the rules and conditions determined by the government of Senegal.

Phase 2 was launched 1 week after Phase 1 (July and August 2011). During Phase 2, 5S practices were implemented at each of the nine locations. Nine days were devoted to this process, which included 1 day at each of the previously established locations. PARSS team members visited the implementation location and provided guidance to the health center staff members in the establishment of 5S practices at the beginning of the day. Staff members subsequently conducted 5S practices for 3–5 consecutive hours under the supervision and onsite guidance of PARSS team members. The activities varied between locations; however, typical ones included cleaning the internal and external spaces, eliminating unwanted items, placing labels and indications, and setting and sorting documents and records. The cost involved in the physical reorganization under the 5S program was nominal; some stationeries and inexpensive tools were purchased with PARSS funds to facilitate 5S practices. No financial incentive was given to the health center staff members during this phase.

During Phase 3, PARSS team members conducted two separate 1-day meetings at the health center to assess the progress of 5S and to provide feedback that could generate further improvements. The first meeting, conducted 1 week after Phase 2, was attended by 43 people, comprising 14 health center staff members, 14 government officials, and 15 external experts and volunteers of JICA. Participation of staff members was limited to those with supervisory functions, including representatives of the nine locations, who were expected to learn the assessment procedure to continue supervising the 5S practice. The participants visited each of the nine locations where the 5S management method had been implemented and assessed the progress. Each participant filled out a ballot that was designed to rank the nine locations from first to ninth in order of their perceived achievement level of the 5S management method. By calculating the means of the ranks for each location, well-performing units were recognized and given prizes of inexpensive items such as office supplies. The participants then held a session to share their observations and to provide advice to the health center staff members on areas for further improvements. Forty-seven people, comprising 14 health center staff members, 21 government officials, and 12 external experts and volunteers of JICA, attended a second meeting in December 2011, 3 months after the first progress-monitoring meeting. The participants assessed the 5S application status in each of the nine locations using an evaluation sheet developed by PARSS team members, and provided feedback for further improvements. The subsistence allowance was paid to the health center staff members who participated in the meetings, but not to those working at the locations that were assessed.

### Individual interview

We conducted data collection for this study in November 2012. To obtain detailed information about staff members’ personal feelings, perceptions, and opinions, we chose face-to-face individual interviews. An external evaluator (the second author [SS], a male Senegalese researcher who holds a PhD in social science and is fluent in English and French), who was not involved in the 5S program and not known to the staff members of the health center, was recruited to conduct the semi-structured interviews. An interview guide was developed in French. To ensure clarity of questions and to gain preliminary insights into a range of potential responses from interviewees, the interview guide was tested with staff members from a different health center in Senegal. We identified interview participants from the staff members of the health center who had participated in both Phase 1 (training and planning) and Phase 2 (5S practice at each unit) of the 5S program based on their availability. During the given period for the interviews, we were able to reach 21 staff members; all of them agreed to participate in the interviews. Participants consisted of 11 men and 10 women (median age: 34 years; range: 25 to 56 years), and they consisted of 9 paramedical staff members, 11 community workers, and 1 support staff member. All the interviews were conducted in French. To guarantee privacy, interviews were conducted in a compartment of the health center where conversations were not audible to other staff members. To avoid bias, each participant was informed before the interview that their responses would be used for research purposes only and would never be used to evaluate the performance of any health center staff members or PARSS team members. The participants did not receive any financial incentives for their participation in the interviews. During the interviews, the participants were asked questions pertaining to changes brought on by the 5S program in the following areas: 1) visible or physical areas of the health center, 2) services provided to patients, 3) their own daily routines, and 4) the work of other health center staff members. Participants were initially asked, in each of these four areas, to indicate a dichotomous answer of ‘Yes’ or ‘No’ to the question as to whether or not they perceived changes after the introduction of the 5S program. They were then asked to elaborate on the nature of the changes they observed. Participants were also prompted to make suggestions for improving the quality of services at the health center. Interview sessions lasted between 20 and 40 min until respondents’ answers were saturated. We digitally recorded the interviews with the permission of participants.

### Data analysis

All interviews were translated from French to English and transcribed to English by the second author (SS, who conducted the interviews). The translated texts were reviewed several times in light of the original interview recordings to ensure that all the information and nuances were adequately converted. The transcribed texts were imported into MAXQDA software, Version 10, and a de-identified data set was prepared to allow thematic analysis (23). The lead author (SK) closely read each transcript several times to become thoroughly familiar with the content and coded the texts to categorize the narrative data into themes. Two of the co-authors (SS and RM) reviewed the coded transcriptions and the themes identified by the first author. All authors discussed disputes and revised the coding categories and themes until consensus was reached. Contradictory views and negative opinions about the 5S program were particularly noted. During this coding process, the identities of the participants were masked to the authors.

### Ethical considerations

We obtained ethical clearance from the National Ethical Committee for Medical Research of the Ministry of Health and Social Action of Senegal and the Research Ethics Committee of the University of Tokyo. Participation in the study was voluntary, and we assured participants of anonymity. In addition, we obtained written consent from each interview participant before each interview. We informed participants that they could withdraw from the study at any point without any risk of sanctions. We used numbers and codes on both the recorded interviews and transcripts to guarantee confidentiality. Furthermore, all problems and constraints identified at the health center under this study were promptly shared with key officials of the Ministry of Health and Social Action.

## Results

From 21 participants’ answers to quantitative questions, we found that a majority perceived that the 5S program brought on changes in each of the following areas: 1) visible or physical areas of the health center (all respondents said ‘Yes’), 2) services provided to patients (Yes=19; No=1; Don't know=1), 3) their own daily routines (all respondents said ‘Yes’), and 4) the work of other health center staff members (Yes=17, No=2, Don't know=2).

We analyzed and classified participants’ narrative responses, and developed a thematic framework that included domains and key themes that were defined based on the responses. We identified four domains that characterized participants’ perspectives of the impact of the 5S program: work environment, attitude and behavior of staff, attitude and behavior of patients, and quality of services. We further subdivided the quality of the service domain into three subdomains: efficiency, patient-centeredness, and safety. Within each domain and subdomain, we identified the key themes (Table 1).

**Table 1:** Key themes identified by staff members about the impact of the 5S program

Domains and sub-domains	Key themes
Work environment	- Fewer unwanted items
	- Improved hygiene and cleanliness
	- Improved orderliness of items
	- Improved labeling and directional indicators of service units
Attitude and behavior	- Increased awareness of 5S
of staff	- Improved collaboration among staff members
	- Increased reuse of items
	- 5S practices extended outside work
Attitude and behavior of patients	- Voluntary participation in maintaining cleanliness of the facility
Quality of services	
Efficiency	- Reduction in time searching for items
	- Improved ability of staff to move around in the office
Patient centeredness	- Reduction in waiting time for patients - Better directions for patients
Safety	- Improved sterilization processes

### Impact on work environment

Participants’ responses pertaining to the work environment domain were represented by key themes, including fewer unwanted items, improved hygiene and cleanliness, improved orderliness of items, and improved labeling and directional indicators of service units (Table 1). Narratives of the participants included: ‘We can easily find drugs to be offered to patients as all the unnecessary items were thrown away’ (Participant L: aged 50–54, male); ‘For instance, at the maternity ward, we no longer confront [the] odor problem …. So, we unanimously recognize that 5S have considerably improved our working environment’ (Participant J: aged 30–34, female); and ‘Yes, I observed that the ticket sellers are more organized, particularly the way they store the money; notes and coins are separated by category’ (Participant B: aged 35–39, male). Because this domain reflects the commonly recognized primary objectives of the 5S program, changes in the work environment reported by participants were direct results of the application of the 5S management method.

### Impact on attitude and behavior of staff

Participants described perceived changes in their own and others’ attitude and behavior after the 5S program implementation. A few participants admitted an increase in their awareness about the principles of 5S; some indicated changes in their attention toward how other people perceived their offices. One participant said, ‘I can say that I am personally motivated to come to work. I am not proud of the past situation where I received visitors in my office with the mess that prevailed’ (Participant P: aged 55–59, male). Another participant answered, ‘Nowadays, the first thing I do in the morning is to free my workplace from garbage and unnecessary items. In fact, I don't want other staff members to find a mess on my working place or desk’ (Participant A: aged 45–49, female).

A few participants indicated that the 5S program brought a culture of recycling and reusing items to the health center. One participant commented about the other office staff members, ‘They don't throw away items as they did before the 5S program. They first think if items are reusable or not. Currently, they recycle many things that used to be usually thrown away’ (Participant D: aged 25–29, female).

Some participants mentioned that collaboration among staff members increased, particularly in educating other staff members on the practice of 5S. Others indicated that they came to practice 5S outside the workplace, such as in their home, because of their exposure to the advantages of the 5S management method in the workplace.

### Impact on attitude and behavior of patients

Some participants noted attitude and behavioral changes in patients after the 5S program was implemented. They stated that the clean environment encouraged patients to maintain the cleanliness of the health center. A participant working at the medical wards reported, ‘The cleanliness of the rooms makes the patients themselves cleaner. We put mops in the rooms and it is not uncommon to see a patient or caretaker cleaning by himself. All this is because of the clean environment’ (Participant Q: aged 40–44, male).

### Impact on quality of services

Participants’ responses indicated changes in the quality of services, particularly in the three subdomains of efficiency, patient-centeredness, and safety, among the six dimensions of healthcare quality proposed by the US Institute of Medicine (24).

#### Efficiency

Almost all the participants mentioned that the 5S program facilitated the identification of items, and hence reduced time spent searching for an item. This efficiency-related measure was raised primarily in the context of the improved orderliness of items in the work environment. A participant in the maternity unit highlighted its impact on service efficiency: ‘Previously we had some difficulties in finding patients’ files following family planning. Since the implementation of the 5S program, we have better organized the workplace by separating out things and clearly indicating the storage sites of documents and files. Nowadays, the family planning consultation is running smoothly. We have also labeled the content of cupboards and shelves; this made it easy to locate documents’ (Participant M: aged 25–29, female).

A few participants mentioned that the ability of the staff to quickly move around the health unit improved in the office following the 5S program. Those responses were mostly attributed to the reduction of unwanted items that had previously prevented staff members from moving around smoothly.

#### Patient-centeredness

About one-half of the participants mentioned reductions in waiting times for patients due to the 5S program. This impact was attributed to improved efficiency at work. One participant remarked, ‘Because documents and files are now in order, we save time ourselves and the patients do not wait so long, unlike before the 5S program’ (Participant D: aged 25–29, female).

About one-half of the participants indicated that it was easier for patients to locate their destination within the health center premises because of the improved labeling and directional indicators of service units. Participants noticed that even slightly literate patients could easily identify the locations to visit because of the improved labeling and directional indicators of service units. In addition, indications of the occupancy of service units better directed patients, as noted by one participant: ‘A sign on the door indicating that the room is occupied was introduced by the 5S program. [Now] patients do not keep on knocking on the door all the time’ (Participant K: aged 35–39, male).

#### Safety

The improvement in the sterilization processes was also attributed to the improved orderliness of items. A participant working at the maternity unit noted, ‘Our working tools are better organized, and the safety has improved because of the systematic sterilization of the medical equipment deriving from the 5S program’ (Participant I: aged 25–29, female).

### Mechanisms of emerging changes

**Fig. 1:**
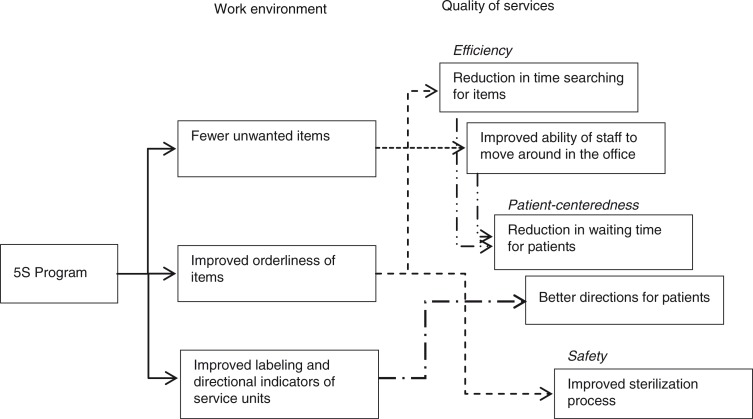
Root cause analysis on perceived changes in the quality of services.

We identified and illustrated the root causes of the perceived changes in the quality of services through a context analysis of the coded transcripts (Fig. 1). The 5S program initially changed the work environment because of fewer unwanted items, improved orderliness of items, and improved labeling and directional indicators of service units. These efforts engendered changes in the quality of services—specifically, making them more efficient, patient-centered, and safe—because of reductions in the time spent searching for items, improved ability to move around in the office, reductions in waiting times for patients, better directions for patients, and improved sterilization processes.

We found that the attitude and behavior changes of staff and patients derived from changes in the work environment and were positively affected by their participation in the 5S program. However, from analysis of participants’ responses under this study, causal relationships were not identified between the improvements in the quality of services and the changes in the attitude and behavior of staff and patients.

### Necessary measures to improve the quality of services at the health center

We coded all 21 interviewees’ suggestions about the necessary measures to improve the quality of services at the health center into 12 categories. Greater physical and material resources were the most frequently mentioned (12 participants), followed by financial incentives for staff members (7), eliminating staff shortages (4), physical arrangements of service units (3), more cleanliness/5S activities (2), and staff training (2). Other measures mentioned included security issues, staff supervision, a drug management system, responsiveness to patients, employment modality, and punctuality.

Among these measures suggested, the limited amount of financial incentives was particularly recognized as a demotivating factor for staff members, as noted by a participant: ‘In order to motivate the staff, the amount of incentives should be improved’ (Participant G: aged 25–29, female). In addition, one participant highlighted the same issue by sharing a negative opinion regarding insufficient incentives given to the health staff members who participated in the 5S program: ‘People complain about PARSS because it asked us to do 5S but did not give substantial incentives …. Recently, we worked with two projects that paid decent subsidies to the community health workers who were involved. You know, the majority of healthcare providers here are community workers, and their wages are very low’ (Participant M: aged 25–29, female). A few participants also suggested better measures to further improve or sustain 5S practice: ‘We need more space for the pharmacy to make 5S practice more visible’ (Participant S: aged 45–49, male); and ‘A routine supervision is also necessary to maintain the good practice of 5S’ (Participant J: 25–29, female).

## Discussion

Through analysis of the interviews, we highlighted a range of changes engendered by the pilot intervention of the 5S management method. We identified that the 5S management method improved the quality of services, and the improvements were rooted in three dimensions: efficiency, patient-centeredness, and safety. Our finding indicated that the improvements in the quality of services were caused by changes in the work environment, including fewer unwanted items, improved orderliness of items, and improved labeling and directional indicators of service units. As with previous studies, no negative impact was perceived about the 5S management method; this could be because it was perceived by nature as a ‘common-sense approach’ (9).

We also identified changes in the attitude and behavior of staff related to the application of the 5S management method. In particular, staff members indicated increased willingness to come to work and efforts toward maintaining a better work environment. From these findings, we suggest that application of the 5S management method contributed to the increase in staff motivation through changes in attitudes and behaviors. Despite the perceived effectiveness of the 5S management method, this study was not designed to assess if the increase in motivation had resulted from the improved status of the work environment, or from the experiences of staff members while participating in the implementation process. Because the 5S management method is an approach that by nature necessitates staff participation in its implementation process, it may not be significantly important for 5S practitioners to identify the degree of contribution of each of these two factors to the motivation increase. Nevertheless, for those designing intervention programs, it might be useful to know if the staff members’ participation in improvement processes could affect staff motivation.

To the best of our knowledge, this is the first study to focus on the application of the 5S management method to a resource-poor facility. Despite the resource constraints faced by the health center, the interviewees suggested that the 5S program led to an improvement in the quality of services. This result, along with the nature of the 5S program as a low-cost and technologically undemanding approach (9), implies that the 5S management method is particularly suitable for improving service quality in resource-poor settings.

The results of our study also implied that the increase in the staff motivation could be brought on by the application of the 5S management method in a resource-poor healthcare facility. Several measures suggested by interviewees for improvement of the quality of services at the health center were directly associated with the motivational factors identified by Willis-Shattuck et al. (25), such as financial incentives, career development, hospital infrastructure, and resource availability. These results indicate that the working conditions at the health center were far from ideal in motivating staff; nevertheless, interviewees indicated that the 5S program led to an increase in staff motivation despite constraints.

The results of several earlier studies indicated associations between the work environment and motivation of health workers in low- and middle-income countries; however, the focus was primarily on the physical infrastructure of healthcare facilities, which is costly (25). Factors related to or efforts toward the orderliness or cleanliness of the workplace, which is attainable at low cost and with little need for technology, were not examined in previous studies. Our study results therefore suggest that improvement of the work environment by the application of the 5S management method possibly could serve as a new approach for motivating staff, particularly in a healthcare facility where resource constraints and other demotivating factors prevail.

In addition, our examination of the implementation process and results of the pilot 5S intervention at the health center contributed to filling knowledge gaps but, at the same time, highlighted areas of further studies and policy implications in the applicability of the 5S management method to healthcare facilities in low- and middle-income countries. First, it was identified that, when the 5S management method is applied to a resource-poor healthcare facility as represented by the health center of our study, its context and roles might be different from those that are usually applied to hospitals in the United States or other healthcare facilities in resource-rich settings. Regardless of workplace settings or situations, the same description is used to depict the 5S management method as the starting point for quality improvement efforts (6–8) . However, where they stand at the starting point could differ depending on the situations of healthcare facilities. Apparently, significant differences would be observed between private hospitals in high-income countries and government healthcare facilities in resource-poor countries as represented by the health center of our study. During the site visits before the pilot intervention, PARSS team members recognized that the work environment at the health center was extremely disorderly; documents and records were piled up or stored in disorganized ways, broken equipment and other unwanted items were kept everywhere unattended, and garbage was scattered around in external spaces. They even found some patient registers dated from 1979 inside a cabinet.

Our study highlighted the potential root causes of the perceived changes in the quality of services engendered by the 5S program (e.g. reduction in time searching for items due to improved orderliness of items, as illustrated in Fig. 1). Considering the initial situation of the health center, these results could alternatively be interpreted as meaning that the extremely disorderly work environment had been a potential bottleneck in providing adequate services. Although variations may exist, we assume that such a work environment is by no means unique to the health center of our study, but can represent many government healthcare facilities managed in a traditional fashion in resource-poor countries. This implies the need to further explore the validity of introducing the 5S management method to those countries, particularly to see if the 5S management method can contribute to removing the bottleneck in providing adequate services at healthcare facilities facing similar challenges.

Second, our study highlighted another area of interest regarding how the 5S management method could contribute to the effective implementation of other quality improvement efforts in such settings. A report based on a case of government hospitals in Tanzania indicated that initial efforts to improve healthcare service quality via a combination of infection control guidelines and continuous quality improvement–total quality management (CQI-TQM) resulted in little progress, and that improvements were seen only after the introduction of the 5S management method (26). To address these issues, the results of our study could be used to develop hypotheses or research questions for further studies as well as to narrow the scope of quantitative studies on the impacts of 5S implementation in such settings.

Third, our study identified a policy implication of its applicability, especially when the 5S management method is applied as a government quality improvement initiative in low- and middle-income countries. As earlier mentioned, the 5S management method has been recognized as a low-cost approach (9). During the pilot intervention at the health center, the cost involved in the physical reorganization was nominal, although some expenses accrued from the organization of the training and meetings. Thus, when the 5S management method is implemented in a single facility, the cost would not be very high or not be much higher than other activities typically conducted in low- and middle-income countries. However, if the goal is to integrate the method into the health system management procedures, additional administrative costs will be incurred in managing an intervention program at a large scale and in ensuring its sustainability. The cost-effectiveness of such an initiative could be of interest among policy makers. This is also another area of further studies that could present policy options to government health authorities in those countries.

This qualitative study was designed to identify possible impacts of the 5S management method from the service provider's perspectives. Needless to say, patients’ viewpoints are also important. We conducted a separate quantitative study to assess the impact of the 5S management method on patient satisfaction. It was conducted at several other health centers where the 5S program was later implemented under PARSS (27).

Several limitations were involved in our study. The interviews were conducted with participants available within the predetermined period of our fieldwork, and additional data collection was not possible. Although study participants were selected indifferently according to their availability during the given timeframe of the study, their responses might not reflect the opinions of all of the staff members of the health center in this study. Last, although an external evaluator (who had not been involved in the intervention and not been known to the participants) was assigned to conduct the interviews, it was not possible to perfectly mask the fact that the data collection was conducted by PARSS, which might have affected their way of giving responses during interviews. It is likely that most of the participants’ statements reflected true information or what they actually perceived; however, exaggerated expressions might have been shared on some occasions.

## Conclusions

The pilot intervention of the 5S management method was perceived to have improved the quality of healthcare services in a resource-poor facility in Senegal. In addition, the improvement of the work environment by the application of the 5S management method was observed to have motivated staff in a healthcare facility where resource constraints and other demotivating factors prevail. Although our results cannot be generalized to other health facilities, they provide a viewpoint for assessing the applicability of the 5S management method, particularly to government healthcare facilities in resource-poor settings where a disorderly work environment serves as a potential bottleneck in providing adequate healthcare services. Quantitative and qualitative research based on a larger-scale intervention would be needed to elaborate and validate these findings as well as to identify the cost-effectiveness of their integration into health systems’ management procedures. The findings of the research can then be used to develop and present policy options, particularly to government health authorities in low- and middle-income countries and representatives of donor agencies that provide support in such fields.
